# Low prevalence of *Plasmodium falciparum *antigenaemia among asymptomatic HAART-treated adults in an urban cohort in Uganda

**DOI:** 10.1186/1475-2875-10-66

**Published:** 2011-03-22

**Authors:** Damalie Nakanjako, Agnes N Kiragga, Barbara Castelnuovo, Daniel J Kyabayinze, Moses R Kamya

**Affiliations:** 1 Department of Medicine, Makerere University School of Medicine, Kampala, Uganda; 2 Infectious Diseases Institute, Makerere University School of Medicine, Kampala, Uganda; 3 Malaria consortium, Kampala, Uganda

## Abstract

**Background:**

Presumptive treatment of malaria is common practice in malaria endemic resource-limited settings. With the changing epidemiology of malaria and the introduction of artemisinin-based combination therapy (ACT), there is increasing need for parasite-based malaria case management to prevent unnecessary use of anti-malarial medicines, improve patient care in parasite-positive patients and identify parasite-negative patients in whom another diagnosis must be sought. Although parasitological confirmation by microscopy or alternatively by malaria rapid diagnostic tests (RDTs) is recommended in all patients suspected of malaria before treatment, gaps remain in the implementation of this policy in resource-limited settings. There is need to evaluate the use of RDTs among highly active anti-retroviral therapy (HAART)-treated people living with HIV (PLHIV).

**Methods:**

Within an urban prospective observational research cohort of 559 PLHIV initiated on HAART and cotrimoxazole prophylaxis between April, 2004 and April, 2005, 128 patients with sustained HIV-RNA viral load < 400 copies/ml for four years were evaluated, in a cross-sectional study, for asymptomatic malaria infection using a histidine-rich protein-2 (HRP-2) RDT to detect *Plasmodium falciparum *antigen in peripheral blood. Patients with positive RDT results had microscopy performed to determine the parasite densities and were followed for clinical signs and symptoms during the subsequent six months.

**Results:**

Of the 128 asymptomatic patients screened, only 5 (4%) had asymptomatic *P. falciparum *antigenaemia. All the patients with positive HRP2 RDT results showed malaria parasites on thick film with parasite densities ranging from 02-15 malaria parasites per high power field. None of the patients with positive RDT results reported signs and symptoms of malaria infection during the subsequent six months.

**Conclusions:**

In an urban area of low to moderate stable malaria transmission, there was low HRP2 P. *falciparum *antigenaemia among PLHIV after long-term HAART and cotrimoxazole prophylaxis. Parasite-based malaria diagnosis (PMD) is recommended among PLHIV that are on long-term anti-retroviral therapy. RDTs should be utilized to expand PMD in similar settings where microscopy is unavailable.

## Background

Presumptive treatment of malaria is common practice among individuals presenting with fever in malaria endemic settings including people living with HIV/AIDS (PLHIV) [1-3]. With the changing epidemiology of malaria and the introduction of artemisinin-based combination therapy (ACT), there is increasing need for parasite-based malaria case management as a strategy to prevent unnecessary use of anti-malarials, improve patient care in parasite-positive patients and identify parasite-negative patients in whom another diagnosis must be sought [4]. Although prompt parasitological confirmation by microscopy or alternatively by malaria rapid diagnostic tests (RDTs) is recommended in all patients suspected of malaria before treatment, gaps remain in the implementation of this policy in resource-limited settings [1,5,6].

HIV-1 infection has been associated with an increased incidence of malaria, and more severe disease [3,7-9]. However, a combination of co-trimoxazole, anti-retroviral therapy, and insecticide-treated bed nets substantially reduced the frequency of malaria in adults with HIV in a rural malaria endemic community [2,8]. There is need to evaluate the use of RDTs among highly active anti-retroviral therapy (HAART)-treated HIV-infected individuals. RDTs have been shown to be over 95% sensitive in detecting *Plasmodium falciparum *antigenaemia [5,10], even in HIV/AIDS rural populations [11,12]. The aim of this study was to determine the prevalence of asymptomatic malaria among HAART-treated patients in an urban malaria meso-endemic setting. The findings emphasize, to HIV/AIDS clinicians, the utility of parasitological-based diagnosis versus presumptive treatment of malaria within HIV treatment programs in sub-Saharan Africa (SSA). This study contributes to the understanding of the potential utility of malaria diagnosis including RDTs among PLHIV in the region.

## Methods

### Study setting and participants

Between April, 2004 and April, 2005, 559 consecutive HAART-naïve HIV-infected adults, were initiated on HAART and enrolled into a prospective observational research cohort as previously described [13] at the Infectious Diseases Institute (IDI) in Kampala, the capital city of Uganda. Patients were initiated on first-line HAART at CD4 counts ≤ 200 cells/μL according to Ugandan guidelines for HAART initiation at the time. Drugs were provided through the Global Fund (a generic combined formulation of stavudine [d4T}, lamivudine [3TC], and nevirapine [NVP] and the US President's Emergency Plan for AIDS Relief (a combined formulation of zidovudine [ZDV] and 3TC plus efavirenz [EFZ] or nevirapine [NVP]. Patients with toxicity to ZDV were changed to tenofovir [TDF]. All patients received cotrimoxazole (or dapsone) prophylaxis according to the national policy to provide cotrimoxazole to all PLHIV. Adherence to HAART was encouraged by at least three individual and group counseling sessions. Patients were reviewed monthly by the study physicians that evaluated among others; adherence to medication, toxicities and acute infections. HIV RNA viral loads, complete blood counts and CD4 counts were measured at six monthly intervals. In addition to cotrimoxazole prophylaxis, all patients receive insecticide-treated bed nets (ITNs) and malaria prevention education as well as prompt treatment of fever symptoms at the study clinic. The patients do not use home-based anti-malarial therapy however they are encouraged to call and/or walk to the study clinic whenever ill. Anti-malarial prescriptions are based on both clinical and microscopy findings and RDTs are not used routinely.

**Figure 1:**
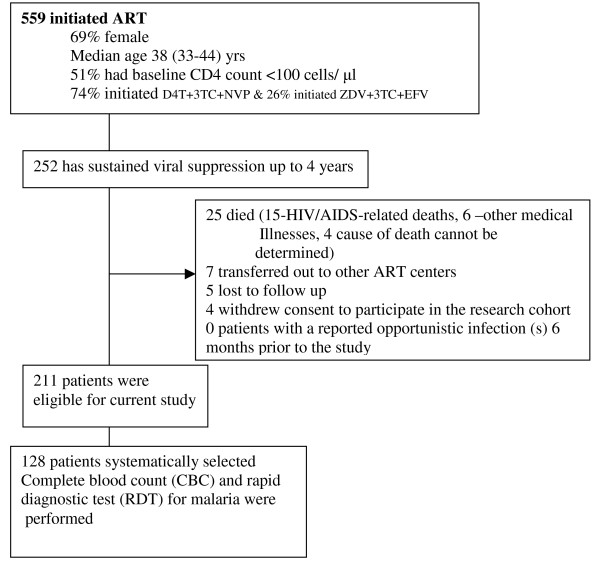
**Profile of patients on anti-retroviral therapy and cotrimoxazole within the Infectious Disease Research cohort**.

After four years of follow up on HAART, 252/559 (45%) patients had sustained HIV-RNA viral suppression defined as HIV-RNA levels < 400 copies/ml within the four years of anti-retroviral therapy. Of these, 41 were excluded due to the following reasons; death (n = 25), lost to follow-up (n = 5), voluntary request to transfer to and voluntary termination from the study (n = 11). Of the remaining 211 patients with chronic HIV infection successfully suppressed on HAART, 128 patients were selected by systematic random sampling (see Figure 1). Every other patient was selected from the register of patients with sustained HIV-RNA viral suppression plus an additional 20% to cater for non-response and loss to follow up. All study participants provided written informed consented and the study was approved by the Uganda National Council For Science and Technology (UNCST).

In a cross-sectional study to evaluate the prevalence of asymptomatic malaria among HAART-treated patients, an RDT was performed for each participant using the P. *falciparum *immuno-chromatographic test (ICT); that detects the *P. falciparum*-specific antigen, histidine-rich protein-2 (HRP-2). The commercially available RDT kit at Uganda health marketing group (UHMG) was used; Diagnostics malaria P. f^® ^cassettes (SSA Diagnostics and Biotech systems, Verna industry estate, Verna, Goa 403722 India) Lot number S21004, manufactured September 2009 and expire August 2011. The manufacturer's storage temperature specifications (4-30°C) were maintained and monitored.

### Laboratory activities

Participants underwent aseptic venipuncture for collection of 3-5 ml of EDTA-anticoagulated whole blood. The haemoglobin level, total white blood cell count and differential counts and the malaria RDTs were performed within 4-6 hrs of sample collection. A drop (5 μl) of whole blood was dropped into the smaller well at one end of the cassette. Five (5) drops of clearing buffer were dropped into the other well. Results were reported within 15 minutes after adding buffer. Appearance of the control band showed that the blood had migrated along the test strip membrane correctly (negative control). A RDT result was interpreted as positive when both the test line and control line showed pink, negative when only the control line showed pink or invalid when the control line did not appear regardless of the test line.

Thick film microscopy was performed, by a trained laboratory technologist, for all the positive HRP2 RDT results. Blood smears were stained with 10% Giemsa for 30 minutes. Thick smears were examined for parasites including gametocytes and parasite densities were determined by counting the number of asexual parasites per 200 white blood cells (WBC), or per 500 WBC if the parasite count was less than 10 parasites per 200 WBC, assuming a WBC count of 8,000/μl. Microscopy was not performed for negative RDT results considering the high specificity of HRP-2 tests in similar settings [12] and the fact that the participants did not have symptoms of malaria illness. Positive thick film results were defined by the presence of one or more confirmed malaria parasite. Participants with positive RDT results were not given anti-malarial drugs considering that they had no symptoms of illness plus the fact that it is not a national policy to treat asymptomatic P. *falciparum *infection. However, these patients were followed up weekly for the first month and monthly for the next five months to determine if they developed signs and symptoms of malaria infection.

## Results

Majority, 94/128 (73%) of the patients were female and the median age was 38 [Interquartile range (IQR), 33-44] years. After four years of HAART with sustained viral suppression, the median CD4 count was 327 (IQR, 247-454) cells/μL. Haematological parameters (haemoglobin, total white cell count and differential counts) were within the normal ranges (see Table 1). Of the 128 patients tested with an RDT, only 5 (4%) had *P. falciparum *antigenaemia. All the patients reported no history of anti-malarial medication within the two months preceding this study. Thick films for patients with positive RDT results showed malaria parasites densities ranging from 02-15 malaria parasites per high power field (hpf) (Table 2). None of the patients with positive RDT results reported signs and symptoms of malaria infection during the subsequent six months.

**Table 1:** Characteristics of 128 patients with sustained viral suppression after 4 years of highly active antiretroviral therapy (HAART) in an urban African cohort

Variable	Parameters 4 years after ART initiation
Age (yrs)	38 (33-44)
Female gender n (%)	94 (73%)
Body mass index (BMI), median (IQR)	22 (20-25)
Baseline CD4 count (cells/μl), median (IQR)	102 (38-167)
CD4 count after 4 years of ART (cells/μl) median (IQR)	327 (247-454)
**HAART regimen**	
Stavudine-Lamividine-Nevirapine/Efavirenz [n (%)]	55 (43%)
Zidovidine-Lamivudine- Nevirapine/Efavirenz [n (%)]	67 (52%)
Tenofovir-Emitricitabine-Nevirapine/Efavirenz [n (%)]	6 (5%)
**Haemogram**	
Total white count, median (IQR)	4429 (3300-5040)
Percentage neutrophils, median (IQR)	44 (37-53)
Percentage lymphocytes, median (IQR)	43(32-47)
Percentage eosinophils, median (IQR)	2 (1-6)
Haemoglobin (mg/dl), median (IQR)	14 (12-15)

**Table 2:** Microscopy results for 5/128 (4%)* asymptomatic patients with Histidine-rich proteins-2 (HRP-2) positive antigenaemia after 4 years of successful highly active antiretroviral therapy (HAART) in an urban African cohort

Patient number	Microscopy results Number of parasites per high power field
1	10 parasites
2	15 parasites
3	05 parasites
4	02 parasites
5	12 parasites

*These patients reported no history of recent treatment of malaria in the previous two months. During follow up, none of the five patients developed clinical symptoms and signs of malaria during the subsequent six months.

## Discussion

This study showed a low prevalence (4%) of asymptomatic *P. falciparum *parasitemia among HAART-treated patients with sustained HIV-RNA viral suppression. The malaria parasite prevalence among HIV patients in this study is lower than the Kampala malaria parasite prevalence of 7.4% among under-five children as reported in the 2009 malaria indicator survey [14]. It is important to note that the latter result is not directly comparable with the current study among HAART & cotrimoxazole-treated adults however it is one of the indicators of malaria endemicity in this urban setting. The findings of this study are consistent with previous reports of low prevalence of malaria infections among successfully treated HIV-infected adults [2,8,15]. All the study participants had received ongoing cotimoxazole prophylaxis, ITNs and malaria prevention education as recommended by the HIV/AIDS treatment guidelines [16]. Results from this study imply that although HIV-infected individuals were previously considered among the special risk groups for malaria infections [3,4], there is need to re-evaluate the risk of malaria infection after long-term successful HAART. The authors recommend parasite-based diagnosis of malaria in similar settings in order to avoid over-prescription of anti-malarial medicines. However, the choice between RDTs and microscopy to implement this depends on local circumstances that include the skills available, patient load, epidemiology of malaria and the possible use of microscopy for the diagnosis of other diseases.

The use of the simple rapid tests to detect malaria antigenaemia among PLHIV emphasizes the utility of RDTs in parasite-based malaria diagnosis (PMD) in this population. The findings of this study will contribute to the implementation of RDTs for PMD among PLHIV in areas where microscopy is not accessible [4]. Moreover, use of RDT results to guide anti-malarial therapy is likely to reduce anti-malarial drug costs due to over-prescription [6,17]. With the introduction of artemesinin-based combination therapy (ACT) [18] coupled with the high pill burden among HIV-infected individuals on HAART, cotrimoxazole and treatment for other co-morbidities, presumptive treatment of malaria is increasingly becoming clinically and economically inappropriate [1]. With the declining incidence of malaria in Africa [1], plus the results from this study, there is need to scale up parasite-based malaria diagnosis in successfully treated HIV-infected adults in areas of low to moderate stable malaria transmission. Similarly, there is need to evaluate the utility of RDTs among successfully treated HIV-infected adults with febrile illnesses.

During follow up, none of the patients with asymptomatic parasitaemia reported development of clinical signs and symptoms of malaria infection during the subsequent six months. It is of interest to note that HIV-infected adults did not develop clinical symptoms of malaria despite the increased risk of malaria infections associated with the HIV-associated immune suppression [3,4,19]. The authors attribute this result partly to the fact that the parasite densities were low since the development of clinical symptoms correlates positively with parasite densities [20]. It is also possible that long-term HAART restores the hosts' ability to control the infection. Similarly, the authors postulate that long-term cotrimoxazole prophylaxis could contribute to the hosts' response to the asymptomatic P. *falciparum *parasitaemia. However, there is need to study the recovery of the host's immunological responses to P. *falciparum *infection among PLHIV in the setting of long-term HAART and cotrimoxazole prophylaxis.

## Conclusion

In an urban area of low to moderate stable malaria transmission, there was low HRP2 P. *falciparum *antigenaemia among PLHIV after long-term successful HAART and cotrimoxazole prophylaxis. Parasite-based malaria diagnosis (PMD) is recommended among PLHIV that are on successful long-term anti-retroviral therapy. RDTs should be utilized to expand the use of PMD in similar settings where microscopy is unavailable.

## Conflict of interest

The authors declare that they have no competing interests.

## Authors' contributions

DN made substantial contribution to the conception, design, data collection, analysis and drafting of the manuscript. ANK contributed to the data analysis, interpretation and revision of the manuscript. BC contributed to the data collection, data interpretation and revision of the manuscript. DJK made substantial contribution to the conception, study design, and interpretation of data. MRK contributed to the conception, design, data interpretation and revision of the manuscript. All authors read and approved the final manuscript.
